# A Comparative Analysis of Cardiac Amyloidosis and Cardiac Sarcoidosis: A Single-Center Experience

**DOI:** 10.3390/jcm14176056

**Published:** 2025-08-27

**Authors:** Luka Katic, Sanjay Sivalokanathan, James Choi, Darren Kong, Vincent A. Torelli, Alexander Silverman, Alexander Nagourney, Usman Saeedullah, Komail Jafri, Syed Zaidi, Serdar Farhan, Ashish Correa

**Affiliations:** 1 Department of Cardiology, Icahn School of Medicine, Mount Sinai Morningside, New York, NY 10025, USA; luka.katic@mountsinai.org (L.K.);; 2 Lenox Hill Hospital, New York, NY 10075, USA

**Keywords:** cardiac amyloidosis, cardiac sarcoidosis, infiltrative cardiomyopathy, echocardiography, cardiac MRI, PET, arrhythmia, heart failure

## Abstract

**Background/Objectives**: Cardiac amyloidosis (CA) and cardiac sarcoidosis (CS) are two distinct infiltrative cardiomyopathies that can present with overlapping clinical features, including heart failure and arrhythmias. However, they arise from fundamentally different pathophysiological mechanisms: amyloid protein deposition in CA versus granulomatous inflammation in CS. These differing pathophysiologies result in divergent imaging patterns, clinical trajectories, and treatment strategies. This study aims to compare the clinical presentations, imaging characteristics, and outcomes of patients with CA and CS to identify key differentiating factors that can improve diagnostic precision and guide therapy. **Methods**: This single-center, retrospective, cross-sectional study analyzed electronic medical records of patients diagnosed with CA (limited to transthyretin CA) or CS at Mount Sinai Morningside system from January 2017 until October 2023. Patients were identified using diagnostic codes and confirmed by histology or disease-specific imaging criteria. Clinical data, transthoracic echocardiography (TTE), cardiac magnetic resonance (CMR) imaging, pyrophosphate scintigraphy (PYP), and fluorodeoxyglucose positron emission tomography (FDG-PET) findings were collected. Statistical comparisons between groups were performed using chi-square tests and independent *t*-tests, with *p* < 0.05 considered statistically significant. **Results**: A total of 16,834 patients were screened and 216 patients were included in the analysis (125 CA, 92 CS). CA patients were older (78.2 vs. 62.0 years, *p* = 0.01), had greater interventricular septal thickness (1.57 vs. 1.10 cm, *p* = 0.01), and exhibited diffuse late gadolinium enhancement (LGE) and elevated extracellular volume (ECV) on CMR. CS patients had higher rates of ventricular tachycardia (53.3% vs. 10.7%, *p* = 0.01), increased myocardial fluorodeoxyglucose (FDG) uptake on positron emission tomography (PET) (90%), and more frequent implantable cardioverter-defibrillator (ICD) placement (66.3% vs. 13.0%, *p* = 0.01). **Conclusions**: CA and CS demonstrate distinct imaging profiles, arrhythmic risks, and treatment patterns. Early differentiation using advanced imaging is crucial for implementing disease-modifying therapies in CA and for immunosuppression and ICD implantation in CS, thereby improving patient outcomes.

## 1. Introduction

Cardiac amyloidosis is a form of infiltrative cardiac disease characterized by the deposition of amyloid fibrils within the myocardial interstitium, which can culminate in a restrictive cardiomyopathy. It may present as heart failure or by manifestations of concomitant systemic amyloidosis, invariably impacting the quality of life and patient survival. Recent advancements in imaging techniques and the development of novel biomarkers have enhanced diagnostic precision, facilitating the differentiation between the two principal forms of cardiac amyloidosis: light-chain amyloidosis (AL) and transthyretin amyloidosis (ATTR). More importantly, treatment modalities have progressed markedly, particularly with the introduction of innovative pharmacological agents that have the potential to modify disease progression in ATTR amyloidosis, as well as targeted chemotherapy options for AL amyloidosis [1]. In contrast, cardiac sarcoidosis is an infiltrative cardiomyopathy resulting from granulomatous inflammation of the myocardium. Although cardiac sarcoidosis (CS) has been noted in only approximately 5% of cases of sarcoidosis, autopsy studies indicate that involvement may be as prevalent as 25% [2]. Common presentations include conduction disease, ventricular arrhythmias, and/or left ventricular dysfunction [2].

The primary objective of this study is to evaluate and compare the clinical presentations, imaging characteristics, and outcomes of patients diagnosed with transthyretin cardiac amyloidosis with those of patients diagnosed with cardiac sarcoidosis. We propose that, despite both CA and CS being “infiltrative diseases” with some overlapping features, there are significant differences in diagnostic findings, clinical outcomes, and responses to treatment strategies.

## 2. Materials and Methods

### 2.1. Study Design, Setting, Patient Enrollment, and Data Collection

This was a retrospective, cross-sectional study. All patients receiving care at the Mount Sinai Morningside cardiology clinic between January 2017 and October 2023 were screened and potential patients were identified from the Electronic Medical Record (EMR) based on diagnostic codes for transthyretin cardiac amyloidosis or cardiac sarcoidosis. Duplicate records were eliminated. For CA, confirmation via histological evidence of amyloid deposits (using Congo red staining) or typical imaging features on PYP scan was required. For CS, the inclusion criteria followed those established by the Heart Rhythm Society (HRS) and the Japanese Circulation Society (JCS), which included evidence from biopsy-proven non-caseating granulomas and supportive cardiac imaging features. Pertinent clinical, laboratory, and imaging data were collected.

### 2.2. Outcome Measures

The primary outcome measures for this study were categorized into three main areas: Imaging Differences, Clinical Outcomes, and Management. Imaging Differences encompassed characteristics from transthoracic echocardiography (TTE) such as ejection fraction, interventricular septal thickness, left ventricular posterior wall thickness, and left atrial volume index, alongside findings from a standardized 1.5 T cardiovascular magnetic resonance (CMR), including MOLLI sequence mapped T1 and extracellular volume (ECV) values and the presence of late gadolinium enhancement (LGE). Additional imaging outcomes from positron emission tomography (PET) scans and PYP scans were also included. Clinical Outcomes focused on the occurrence of atrial fibrillation, atrial flutter, ventricular tachycardia and the placement of implantable cardioverter-defibrillators (ICD). Lastly, Management examined the initiation of disease-specific treatments, specifically tafamidis for transthyretin amyloid cardiomyopathy and various forms of immunosuppression for sarcoidosis. Very recently introduced disease-modifying therapies for transthyretin amyloid cardiomyopathy, such as acoramidis and vutrisiran, were omitted.

### 2.3. Statistical Analysis

Data were analyzed using IBM SPSS Statistics for Windows, version 30 (IBM Corp., Armonk, NY, USA). Continuous variables are presented as means ± standard deviations, and categorical variables as counts and percentages. Chi-square tests were used to examine associations between categorical variables, and independent samples *t*-tests were used to compare continuous variables between groups. A significance level of *p* < 0.05 was set for all tests.

### 2.4. Missing Data

The study employed a complete-case analysis approach due to its retrospective design; therefore, only variables with complete data were included in the final analyses. For primary outcomes and critical imaging data, missing data did not exceed 5%, ensuring robustness and validity of the analysis. The retrospective design ensured there were no losses to follow-up.

## 3. Results

### 3.1. Demographics

A total of 16,834 patients received care at the Mount Sinai Morningside cardiology clinic from January 2017 to October 2023. Of these, 216 patients were included in the analysis (125 with CA and 92 with CS). The demographic analysis of the study population revealed statistically significant differences in several variables between patients with cardiac amyloidosis and those with cardiac sarcoidosis. The mean age for patients with cardiac amyloidosis was significantly higher at 78.2 years compared to 62.0 years for those with sarcoidosis (*p* = 0.01). Rates of smoking were notably lower in the sarcoidosis group (18.5%) compared to the amyloidosis group (36.8%), showing a significant difference (*p* = 0.01). Hypertension was more prevalent among amyloidosis patients (87.2%) than sarcoidosis patients (75.0%), with a statistically significant *p*-value of 0.02. Significant differences were also observed in the family history of disease, with 24.8% of amyloidosis patients reporting a family history, compared to only 5.4% in the sarcoidosis group (*p* = 0.01). Additionally, significant disparities were found in previous stent placement (23.2% in amyloidosis vs. 5.4% in sarcoidosis, *p* = 0.01) and the prevalence of heart failure (93.6% in amyloidosis vs. 68.5% in sarcoidosis, *p* = 0.01). No significant differences were found in sex distribution, body mass index, diabetes mellitus, hyperlipidemia, myocardial infarction, and history of coronary artery bypass grafting (CABG) between the two groups. (Table 1).

### 3.2. Imaging Results

Imaging comparisons between CA and CS groups revealed notable differences across several modalities. In transthoracic echocardiography (TTE), the interventricular septal (IVS) thickness and left ventricular posterior wall thickness (LVPWd) were significantly greater in cardiac amyloidosis patients, with mean thicknesses of 1.57 ± 0.35 cm and 1.45 ± 0.34 cm, respectively, compared to 1.10 ± 0.25 cm and 1.04 ± 0.18 cm in sarcoidosis patients (*p* = 0.01 for both). Conversely, there was no significant difference in ejection fraction (EF) and left atrium (LA) volume index between the groups. Mitral regurgitation (MR) both moderate and severe, was observed more often in the amyloidosis group than the sarcoidosis group, however only moderate MR returned a statistically significant difference (36 vs. 14, *p* = 0.03).

In CA, of the 19 out of 125 patients who underwent CMR, the results showed a mean Native T1 mapping value of 1119 ± 59 ms and an ECV of 56.9 ± 14.7%, indicating significant myocardial infiltration. This was further corroborated by a 100% rate of diffuse LGE on CMR. PYP highlighted extensive amyloid deposition, with 73 (58.4%) patients displaying high uptake (Grade 3) and 22 (17.6%) showing moderate uptake (Grade 2).

Conversely, in the cardiac sarcoidosis group, out of 92 patients, 77 had CMR, with 51 (66%) showing LGE. PET was performed in 80 patients, where intense fluorodeoxyglucose (FDG) uptake was noted in 72 (90%) of the cases, and a ‘mismatch pattern’ (FDG uptake in the same areas as perfusion defects) was identified in 40 (50%) of these patients. (Table 2).

### 3.3. Outcome

The outcome analysis for patients with cardiac amyloidosis and cardiac sarcoidosis shows significant differences in clinical endpoints, which may reflect the underlying pathophysiological differences between these two conditions. Atrial fibrillation was more common in cardiac amyloidosis (57.6%) compared to cardiac sarcoidosis (45.7%), though the difference was not statistically significant (*p* = 0.08). However, significant disparities were observed in other areas: atrial flutter occurred in 31.2% of amyloidosis patients versus 17.4% of sarcoidosis patients (*p* = 0.02); ventricular tachycardia was markedly higher in the sarcoidosis group, at 53.3%, compared to 10.7% in the amyloidosis group (*p* = 0.01). The placement of implantable cardioverter-defibrillators (ICDs) was significantly more common in patients with cardiac sarcoidosis (66.3%) than in those with cardiac amyloidosis (13.0%), possibly reflecting a greater incidence of life-threatening arrhythmias in sarcoidosis (*p* = 0.01). (Table 3).

### 3.4. Therapy

The analysis of management strategies between the CA and CS groups demonstrated significant differences in treatment approaches, reflecting the distinct pathophysiology of these conditions. In cardiac amyloidosis, a substantial majority of the patients, 70.4% (88 out of 125), were treated with Tafamidis, a medication specifically targeting transthyretin stabilization to mitigate the progression of amyloidosis. Conversely, in the cardiac sarcoidosis group, 80.4% (74 out of 92) received any immunosuppressive therapies. In comparison, 57.6% (53 out of 92) remained on chronic/steroid-sparing immunosuppression, the therapeutic standard in managing the inflammatory aspects of sarcoidosis. While narrowing the threshold for statistical significance, this data still highlights the need for tailored therapeutic strategies to address the specific clinical manifestations and disease mechanisms inherent to each of these severe cardiac conditions (Table 4).

## 4. Discussion

Differentiating cardiac amyloidosis from cardiac sarcoidosis is of critical clinical importance, as both fall under the umbrella of infiltrative cardiomyopathies but represent fundamentally distinct pathophysiological processes with divergent clinical manifestations, imaging profiles, and disease trajectories. CA is characterized by the extracellular deposition of amyloid fibrils, leading primarily to restrictive physiology and left ventricular hypertrophy [1]. In contrast, CS is driven by granulomatous inflammation, which predominantly results in conduction abnormalities and ventricular arrhythmias, without producing significant LVH on imaging [2].

These disorders are positioned at different points along the spectrum of cardiomyopathy. Though they are often clubbed together by virtue of the “infiltrative” pathophysiologic bases, they are very different conditions: CA often presents as a progressive, infiltrative disease that progresses to a restrictive cardiomyopathy (hypertrophied myocardium with severe diastolic dysfunction), while CS behaves more as an inflammatory disorder with a high arrhythmic burden and only a portion of these patients progress to dilated or restrictive cardiomyopathies [3,4]. Moreover, their natural histories and prognoses are shaped by their underlying pathophysiology; untreated CA can rapidly progress to refractory heart failure, whereas CS has a waxing and waning clinical course with episodes of inflammation and clinical quiescence between these episodes [5,6].

Given these differences, early and accurate differentiation is essential not only for prognostication but also for guiding therapy. CA management centers on amyloid-directed therapies, such as tafamidis (for ATTR) or chemotherapy (for AL), whereas CS requires immunosuppressive agents to curb granulomatous myocardial inflammation and prevent arrhythmic complications [1,2]. Therefore, recognizing the unique clinical trajectories, imaging hallmarks, and treatment needs of CA and CS is paramount for clinicians aiming to improve patient outcomes in these high-risk populations.

### 4.1. Demographics Discussion

Our cohort demonstrated a significantly older age among patients with cardiac amyloidosis (78.2 ± 11.3 years) compared to those with cardiac sarcoidosis (62.0 ± 11.9 years), consistent with previous reports that document advanced age distributions in amyloid populations, particularly among male patients [7,8]. In contrast, cardiac sarcoidosis cohorts, such as the Finnish registry, have described a higher proportion of females, underlining demographic variability in sarcoidosis that can be influenced by sex and race [9,10,11]. Although the body mass index (BMI) did not differ significantly between the two groups (27.7 ± 5.8 vs. 29.0 ± 5.6, *p* = 0.13), the clinical relevance of BMI in either disease remains unclear, given that disease severity and organ involvement are often more critical factors [7].

Hypertension was significantly more frequent in the amyloidosis cohort (87.2%) compared to the sarcoidosis cohort (75.0%), aligning with existing literature that suggests elevated blood pressure in amyloidosis may indicate a more severe phenotype [12]. Nonetheless, other studies point out that specific cardiac amyloidosis subsets display lower rates of hypertension, signifying heterogeneous clinical presentations [13]. For sarcoidosis, our findings resonate with pooled analyses noting considerable rates of hypertension (28.8%) and an elevated risk of heart failure, both underscoring the complexity of managing cardiac sarcoidosis [14,15]. Long-term corticosteroid use may further compound metabolic issues, contributing to hypertension and diabetes mellitus [16]. Smoking habits also differed notably, with fewer sarcoidosis patients (18.5%) being smokers than those with amyloidosis (36.8%), which aligns with studies indicating that nonsmokers might exhibit a higher degree of myocardial inflammation in cardiac sarcoidosis [17]. Meanwhile, hyperlipidemia rates were similar (70.4% vs. 71.7%), although aberrant lipid profiles such as Lipoprotein-X in sarcoidosis highlight the need for meticulous lipid evaluation [18].

Family history was more prevalent in the amyloidosis group (24.8% vs. 5.4%), consistent with epidemiological data on variant transthyretin (ATTR) amyloidosis, particularly the Val122Ile mutation, which is more commonly seen in African American and Caribbean descendants [19]. In contrast, sarcoidosis presents a more intricate genetic landscape, with ethnic and familial predispositions that are not seemingly straightforward but do contribute to disease vulnerability, particularly in Northern Europeans and African Americans [20]. Some patients initially labeled with “isolated cardiac sarcoidosis” have indeed been discovered to harbor pathogenic variants of inherited cardiomyopathies, emphasizing the significance of genetic testing in ambiguous cases [21].

Interestingly, prior coronary interventions (e.g., stent placement) also differed: 23.2% in amyloidosis versus 5.4% in sarcoidosis, although myocardial infarction (10.4% versus 7.6%) and CABG (6.4% versus 3.3%) were relatively rare in both groups. We believe this difference is primarily influenced by the age disparity between the groups, which is consistent with findings from national databases. The National Readmissions Database, which includes 4252 CA patients, shows a mean age of 73.3 ± 11.7 years, with approximately 10% having a history of STEMI. The post-STEMI period was associated with high rates of multi-organ complications, regardless of whether patients underwent PCI or CABG, ultimately resulting in similar mortality outcomes among CA-STEMI patients irrespective of intervention [22,23].

### 4.2. Imaging Discussion

**Figure 1:**
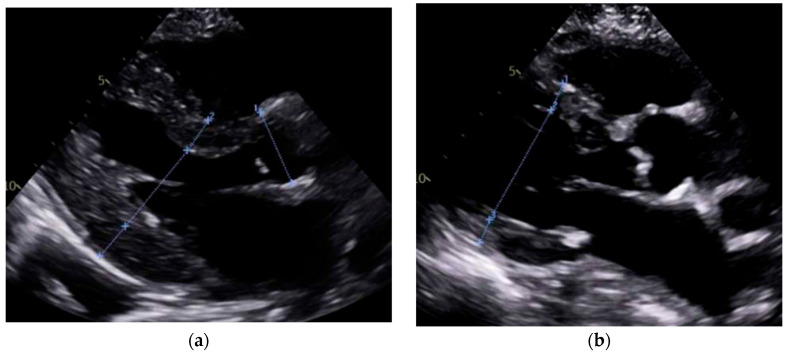
**Parasternal long-axis transthoracic echocardiography (TTE) comparison.** (**a**) Cardiac amyloidosis showing diffuse concentric left ventricular wall thickening and sparkling myocardial texture, with a small left ventricular cavity and biatrial enlargement. (**b**) Cardiac sarcoidosis showing normal to mildly thickened walls, with possible basal septal thinning or regional motion abnormalities, and preserved left ventricular cavity size.

**Figure 2:**
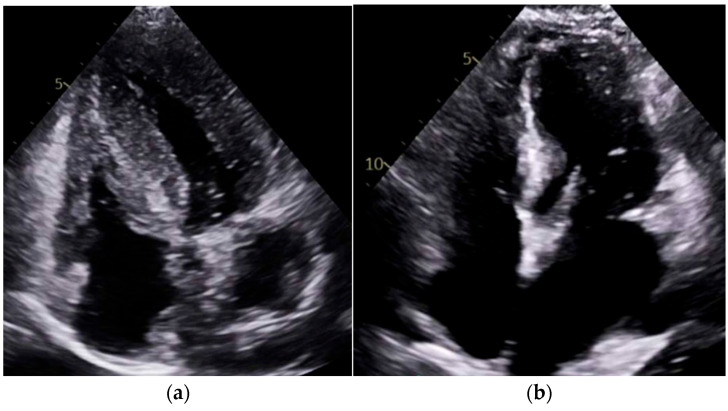
**Apical four-chamber transthoracic echocardiography (TTE) comparison.** (**a**) Cardiac amyloidosis is characterized by thickened ventricular walls, small ventricular chambers, and biatrial enlargement, consistent with restrictive cardiomyopathy. (**b**) Cardiac sarcoidosis showing relatively normal wall thickness, patchy wall thinning (often basal septum), and regional motion abnormalities, with atrial enlargement.

**Figure 3:**
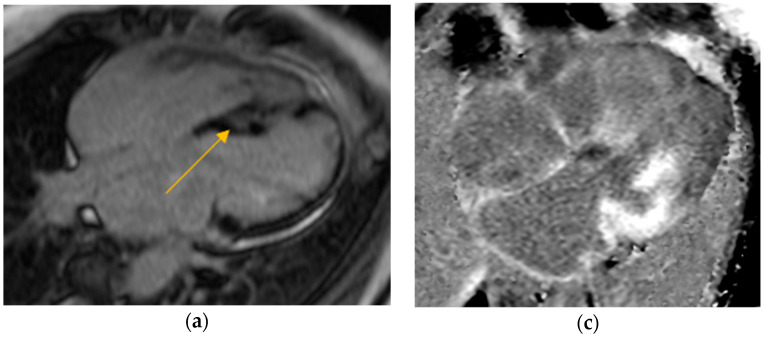

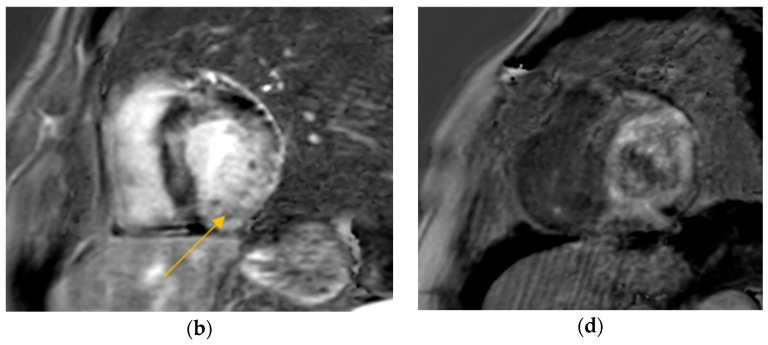
**Cardiac MRI examples of late gadolinium enhancement (LGE).** (**a**) Four-chamber view showing septal LGE (scar). (**b**) Short-axis view demonstrating a large inferolateral scar. Both (**a**,**b**) demonstrate the patchy LGE pattern characteristic of cardiac sarcoidosis, marked by the yellow arrow. (**c**) Four-chamber view and (**d**) short-axis view showing diffuse subendocardial and transmural LGE in a global distribution, consistent with cardiac amyloidosis.

**Figure 4:**
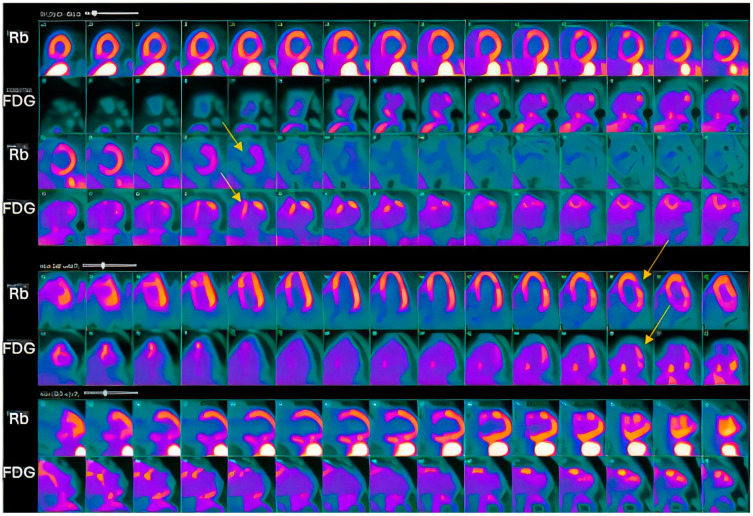
**FDG PET Imaging** cardiac sarcoidosis demonstrating a perfusion–metabolism mismatch pattern, as marked by yellow arrows.

Our study found significantly greater IVSd and LVPWd in amyloidosis compared to sarcoidosis, consistent with prior reports on echocardiographic findings in amyloidosis namely, pronounced LV wall thickening and diastolic dysfunction (Figure 1 and Figure 2). Ref [24], Conversely, TTE offers limited sensitivity in sarcoidosis, functioning mainly to prompt further diagnostic imaging when regional wall thinning or basal septal changes are detected [25]. Our CMR findings revealed focal or patchy LGE in sarcoidosis, as well as mismatch uptake patterns on PET, which parallels the existing literature that utilizes CMR for structural analysis and FDG PET for monitoring active inflammation [26,27]. Our findings in the CA cohort corroborate that PYP scintigraphy (with a negative monoclonal protein screen) remains highly specific for transthyretin cardiac amyloidosis [28]. Additionally, all 19 of our CA patients undergoing CMR exhibited a diffuse pattern of LGE, highlighting the global amyloid infiltration of myocardium in contrast to the patchy granulomatous infiltration in CS. More specific measures on CMR such as Native T1 mapping and ECV measurements can indicate the extent of amyloid infiltration [29]. Unfortunately, only 19 of the 125 amyloidosis patients underwent CMR in our study, which limited the robustness of sub-analyses in this domain (Figure 3 and Figure 4).

### 4.3. Outcome Discussion

Atrial fibrillation (AF) occurred more commonly in amyloidosis (57.6%) than in sarcoidosis (45.7%), aligning with previously reported AF rates approaching 70% in wild-type transthyretin amyloidosis [30]. As such, anticoagulation and rhythm control agents are often adjuncts to standard amyloidosis management, regardless of CHA_2_DS_2_-VASc scores; however, catheter ablation carries a high risk of recurrence [31,32]. In sarcoidosis, AF has been increasingly linked to direct atrial inflammation, especially when FDG PET shows atrial uptake [33]. Immunosuppression can reduce the inflammatory component, although its effectiveness in purely atrial arrhythmias remains uncertain [34]. Atrial flutter may also stem from direct sarcoid involvement of the atria, mandating immunosuppressive therapy and potential ablation [35].

In contrast, the rates of ventricular tachycardia (VT) (53.3% vs. 10.7%) and ICD placement (66.3% vs. 13.0%) were significantly higher in sarcoidosis than in amyloidosis, which is consistent with the recognized prevalence of ventricular arrhythmias in infiltrative cardiomyopathies [36]. While AL amyloidosis carries a significant risk of ventricular arrhythmias, and one population-based study noted a 153-fold increase in ventricular tachycardia, the utility of ICDs is frequently limited by electromechanical dissociation and atrioventricular (AV) block [37,38]. Sarcoidosis, though more prone to VT, benefits from timely ICD implantation, likely explaining why higher VT rates do not translate into higher mortality [39].

## 5. Conclusions

Collectively, these findings highlight the critical importance of distinguishing between cardiac amyloidosis and sarcoidosis in clinical practice, given the significant differences in age distributions, imaging signatures and arrhythmia profiles. Utilizing advanced imaging modalities, such as CMR, PYP, and PET, to identify hallmark disease features, including TTR-specific uptake or inflammatory mismatch patterns, can inform individualized treatment strategies, including disease-modifying agents for ATTR amyloidosis or immunosuppression for sarcoidosis. However, our single-center, cross-sectional analysis relies on limited CMR data and a small sample size. Future research and larger datasets could validate our observed associations, refine arrhythmic risk stratification, explore disease-specific genetic underpinnings, and assess emerging therapeutics, such as vutrisiran and acoramidis in amyloidosis, and novel immunomodulators in sarcoidosis.

In conclusion, our study demonstrates key demographic, imaging, and outcome-related distinctions between CA and CS. Greater awareness of these distinctions will ultimately enhance diagnostic precision, inform tailored therapeutic approaches, and potentially improve patient outcomes in both of these challenging cardiac conditions.

## Figures and Tables

**Table 1:** Demographic analysis.

Variable	Cardiac Amyloidosis (*n* = 125)	Cardiac Sarcoidosis (*n* = 92)	*p* Value *
Age (years)	78.2 ± 11.3	62.0 ± 11.9	0.01
Sex (Male)	85 (68.0%)	61 (66.3%)	0.79
BMI (kg/m^2^)	27.7 ± 5.8	29.0 ± 5.6	0.13
Hypertension (Yes)	109 (87.2%)	69 (75.0%)	0.02
Diabetes Mellitus (Yes/No)	49 (39.5%)	27 (29.3%)	0.12
Smoking (Yes/No)	46 (36.8%)	17 (18.5%)	0.01
Hyperlipidemia (Yes/No)	88 (70.4%)	66 (71.7%)	0.83
Family History of Disease	31 (24.8%)	5 (5.4%)	0.01
Myocardial Infarction	13 (10.4%)	7 (7.6%)	0.48
Previous Stent Placement	29 (23.2%)	5 (5.4%)	0.01
History of CABG	8 (6.4%)	3 (3.3%)	0.36
Heart Failure	117 (93.6%)	63 (68.5%)	0.01

* Result of the statistical test of choice (Chi square vs. *t*-test).

**Table 2:** Cardiovascular imaging differences.

Imaging Modality	Parameter	Cardiac Amyloidosis	Cardiac Sarcoidosis	*p* Value
TTE		(*n* = 124)	(*n* = 87)	0.70
	EF (%)	48 ± 15	49 ± 14	
	IVSd (cm)	1.57 ± 0.35	1.10 ± 0.25	0.01
	LVPWd (cm)	1.45 ± 0.34	1.04 ± 0.18	0.01
	LAVi (mL/m^2^)	45.77 ± 14.07	31.48 ± 14.57	0.75
	MR	Moderate [36 (29%)]	Moderate [14 (16.1%)]	0.03
		Severe [8 (6.5%)]	Severe [1 (1.1%)]	0.08
CMR		(*n* = 19)	(*n* = 51)	
	Native T1 Mapping (ms)	T1—1119 ± 59	N/A	
	Extracellular Volume (%)	ECV—56.9 ± 14.7	N/A	
	LGE	Diffuse—(100%)	Patchy—(66%)	0.01
PYP	PYP Scale	Grade 2—22 (17.6%)	N/A	
		Grade 3—73 (58.4%)		
PET	FDG Uptake (SUV)	N/A	72/80 (90%)	
	PET Mismatch Pattern		40/80 (50%)	

TE: Transthoracic Echocardiography; EF: Ejection Fraction; IVSd: Interventricular Septum thickness in diastole; LVPWd: Left Ventricular Posterior Wall thickness in diastole; LA: Left Atrium; LAVi: Left Atrial Volume index; MR: Mitral Regurgitation; CMR: Cardiac Magnetic Resonance Imaging; ECV: Extracellular Volume; LGE: Late Gadolinium Enhancement; PYP: Pyrophosphate Scintigraphy; PET: Positron Emission Tomography; FDG: Fluorodeoxyglucose. *p*-values are from Chi-square or *t*-test as appropriate.

**Table 3:** Outcome analysis.

Variable	Cardiac Amyloidosis (*n* = 125)	Cardiac Sarcoidosis (*n* = 92)	*p* Value
Atrial Fibrillation	72 (57.6%)	42 (45.7%)	0.08
Atrial flutter	39 (31.2%)	16 (17.4%)	0.02
Ventricular Tachycardia (VT)	13 (10.7%)	49 (53.3%)	0.01
Implantable Cardioverter-Defibrillator (ICD) Placement	16 (13.0%)	61 (66.3%)	0.01

Result of the statistical test of choice (Chi square vs. *t*-test).

**Table 4:** Demographic analysis.

	Cardiac Amyloidosis	Cardiac Sarcoidosis	*p* Value
Management	Tafamidis (88, 70.4%)	Immunosuppression (74, 80.4%) Steroid Sparing Therapy (53, 57.6%)	0.093 0.051

Result of the statistical test of choice (Chi-square test).

## Data Availability

All data supporting the findings of this study are available from the corresponding authors upon reasonable request.

## Abbreviations

The following abbreviations are used in this manuscript:

CA	Cardiac Amyloidosis
CS	Cardiac Sarcoidosis
AL	Light-Chain Amyloidosis
ATTR	Transthyretin Amyloidosis
LGE	Late Gadolinium Enhancement
ECV	Extracellular Volume
ICD	Implantable cardioverter-defibrillator
PYP	Pyrophosphate Scintigraphy
FDG-PET	Fluorodeoxyglucose Positron Emission Tomography
